# Reduced L-Arginine and L-Arginine-ADMA-Ratio, and Increased SDMA after Norseman Xtreme Triathlon

**DOI:** 10.3390/sports9090120

**Published:** 2021-08-31

**Authors:** Christoffer Nyborg, Martin Bonnevie-Svendsen, Helene Støle Melsom, Jørgen Melau, Ingebjørg Seljeflot, Jonny Hisdal

**Affiliations:** 1 Institute of Clinical Medicine, Faculty of Medicine, University of Oslo, 0318 Oslo, Norway; martin.bonnevie@gmail.com (M.B.-S.); h.s.melsom@studmed.uio.no (H.S.M.); jorgen@melau.no (J.M.); uxinlj@ous-hf.no (I.S.); jonny.hisdal@medisin.uio.no (J.H.); 2 Department of Vascular Surgery, Oslo University Hospital, 0424 Oslo, Norway; 3 Department of Prehospital Care, Vestfold Hospital Trust, 3103 Toensberg, Norway; 4 Center for Clinical Heart Research, Department of Cardiology, Oslo University Hospital, 0424 Oslo, Norway

**Keywords:** L-arginine, ADMA, SDMA, NO, ironman, triathlon

## Abstract

Endothelial vasodilatory function is dependent on the NO synthesis from L-arginine by endothelial NO-synthetase (eNOS). eNOS can be inhibited by asymmetric dimethylarginine (ADMA) by competitive inhibition on the binding site, and symmetric dimethylarginine (SDMA) can reduce the L-arginine availability intracellularly through competing for transport over the cellular membrane. To study the NO synthesis after prolonged exercise, we assessed circulatory L-arginine, the L-arginine/ADMA ratio, and SDMA before, after, and on the day after the Norseman Xtreme triathlon, an Ironman distance triathlon. We found significantly reduced levels of L-arginine and the L-arginine/ADMA ratio and increased levels of SDMA after the race (all *p* < 0.05). L-arginine rose toward baseline levels the day after the race, but ADMA increased beyond baseline levels, and SDMA remained above baseline the day after the race. The reduced levels of L-arginine and the L-arginine/ADMA ratio, and increased SDMA, after the race indicate a state of reduced capability of NO production. Increased levels of ADMA and SDMA, and reduced L-arginine/ADMA ratio, as seen the day after the race, are known risk markers of atherosclerosis and warrant further studies.

## 1. Introduction

Prolonged exercise is known to induce many physiological changes, including alteration of the circulatory inflammatory biomarkers [1,2,3,4] and plasma levels of essential nutrients [5]. Our research team found a transient reduction in endothelial function in a recent study after the Norseman Xtreme triathlon, an ironman distance triathlon. Endothelial function was measured with flow-mediated dilation (FMD) [6]. FMD is measured by studying brachial artery dilatation in response to increased blood flow after a 5 min occlusion of the lower arm at rest [7]. The dilatation is dependent on the endothelium [8], as increased share forces from the blood flow activate mechanoreceptors in the endothelium [9], causing a predominantly NO-mediated relaxation of the arterial smooth muscles [10].

NO is synthesized by the endothelial NO-synthetase (eNOS) from the amino acid L-arginine [11,12]. In our previous study, we also found reduced levels of circulatory L-arginine after completion of the Ironman triathlon [6]. Previous studies of amino acids after exercise have shown a generally reduced level of amino acid-bound nitrogen in prolonged exercise [13] and reduced L-arginine after marathons [14,15]. This reduction is believed to be due to increased gluconeogenesis and enhanced NO production during prolonged exercise [13,14]. Thus, low levels of L-arginine could, in part, explain the reduced endothelial function after prolonged exercise, as described in our previous study [6]. 

In recent years, there has been a growing interest in the methylated forms of L-arginine: asymmetric dimethylarginine (ADMA) and symmetric dimethylarginine (SDMA), regarding their effect upon NO synthesis [16]. ADMA reduces the activity of eNOS through competitive inhibition on the binding site for L-arginine [17,18]. Therefore, the L-arginine/ADMA ratio is of importance for NO synthesis [19]. SDMA has no direct effect on eNOS [17], but there is evidence that SDMA has a weak indirect inhibition of NO synthesis [20] and L-arginine transport [21]. Elevated levels of both ADMA and SDMA have both been associated with all-cause mortality and cardiovascular death [22]. 

Studies of the effect of prolonged exercise on circulating ADMA and SDMA are scarce. One recent study of 14 athletes that completed a 100 km run found elevated levels of SDMA and unchanged levels of ADMA [23]. However, they did not measure L-arginine and did not calculate L-arginine/ADMA ratio, making it difficult to interpret the significance of the results for NO synthesis. Furthermore, there are no studies to our awareness on ADMA, SDMA, and L-arginine after an Ironman distance triathlon.

To further elucidate the NO synthesis after prolonged exercise, we analyzed serum samples from the Norseman Extreme triathlon 2019 for L-arginine, ADMA, and SDMA, and calculated the L-arginine/ADMA ratio before, after, and one day after the race. We hypothesized that L-arginine was transiently reduced after the race and would normalize the day after. Based on our previous findings, we further hypothesized the L-arginine/ADMA ratio to be reduced. Further, we aimed to explore the changes in levels of ADMA and SDMA after prolonged strenuous exercise. 

## 2. Materials and Methods

### 2.1. Study Population

Participants in the Norseman Xtreme Triathlon 2019 were recruited through email invitation before the race. Willing participants were given information about the study and signed the informed consent. Forty participants who met for baseline measurements the day before the race and completed the race were included. The participants had blood samples taken at noon the day before the race, as soon as possible after crossing the finish line, and at noon the day after the race. 

The Regional Committee for Medical and Health Research Ethics in Norway (REC) approved all experimental measurements (reference: 2016/932), and the study was conducted according to the Declaration of Helsinki. 

### 2.2. Blood Samples

Venous blood samples were collected in vacuum containers with silica particles and gel separators. The first sample was taken within 24 h before race start, the second sample immediately after race completion, and the third sample at noon the day after the race. The blood was clotted for 30 min at room temperature, serum was separated by centrifugation at 2000× *g* for 10 min within 1 h, and the serum was pipetted to separate freeze-tolerant containers. All samples were transported on ice to freezing storage with a temperature of −80 °C and was analyzed ten months later by high-performance liquid chromatography (HPLC) and precolumn derivatization with o-phthaldialdehyde (OPA) (Sigma Chemicals Co, St Louis, MO, USA) for levels of L-arginine, ADMA, and SDMA (coefficients of variation: L-arginine 5.6%; ADMA 7.0%; SDMA 9.6%). The L-arginine/ADMA ratio was calculated for each time point.

### 2.3. Statistics and Visualization

One-way repeated ANOVA with timepoints as within-factor was conducted to test for significant changes in L-arginine, ADMA, SDMA, and the L-arginine/ADMA ratio. Missing data were assumed to be completely random, and ANOVA was conducted on the complete sets of three samples. Post hoc paired *t*-tests with Bonferroni corrections were used to test differences between the time points on all available measurements. A *p*-value of <0.05 was considered significant. All values are presented as mean ± SD. Statistics were conducted, and graphs were created in R (R version 4.0.3, R Foundation for Statistical Computing, Vienna, Austria). Figure 2 was created with an online application from biorender.com (BioRender, Toronto, ON, Canada).

## 3. Results

### 3.1. Subjects

Forty participants of the Norseman Xtreme Triathlon were included in this study. All subjects completed the race. Characteristics of the subjects are given in Table 1.

### 3.2. Biomarkers

Mean values with standard deviations are given for all the variables in Table 2. L-arginine and ADMA were significantly reduced at the end of the race, with a reduced L-arginine/ADMA ratio. Both plasma levels of L-arginine and ADMA inclined to the day after the race. L-arginine did not incline fully to baseline levels, while ADMA inclined beyond baseline levels the following day, causing an even lower L-arginine/ADMA ratio the following day than right after the finish. 

SDMA was elevated compared to baseline levels after the race, and there were similar elevated SDMA plasma levels the day after the race without significant changes from the finish line. 

**Figure 1:**
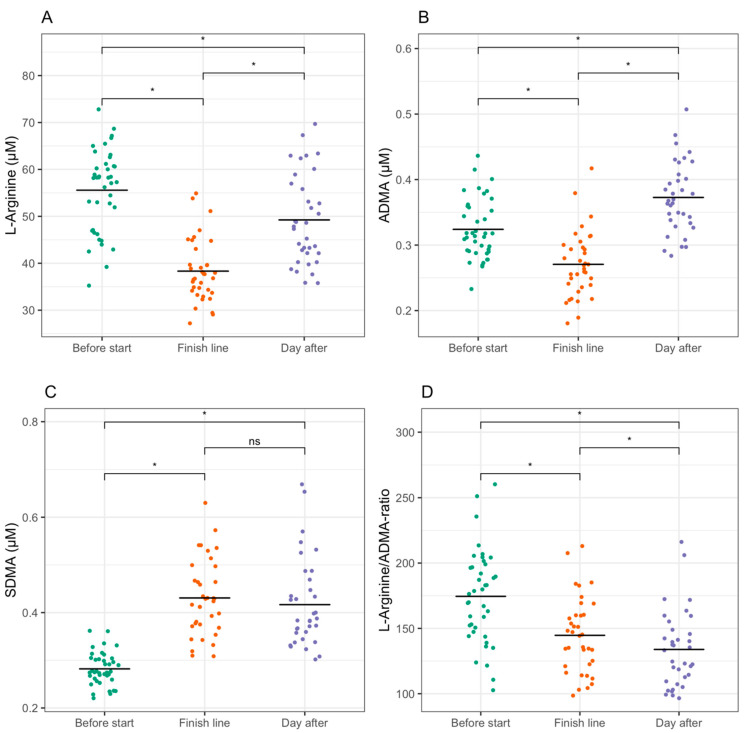
Individual levels of L-arginine (**A**), ADMA (**B**), SDMA (**C**), and L-arginine/ADMA ratio (**D**) before the start, at the finish line, and the day after the race with results from two-sided post hoc paired *t*-tests with Bonferroni corrections. * Indicates *p*-value < 0.05, and ns indicates *p*-value > 0.05. The bold lines indicate mean values for each measurement. Repeated-measures ANOVA tests for all the biomarkers showed *p*-value < 0.05.

Individual values and results from the statistical test are shown in Figure 1. Raw data with individual values are available in the Supplementary materials.

## 4. Discussion

The main finding in the present study was that the levels of L-arginine and the L-arginine/ADMA ratio were reduced immediately and the day after an Ironman distance triathlon. The reduction in L-arginine/ADMA ratio has, to our knowledge, not been previously reported after any Ironman triathlon or other prolonged exercise events. 

**Figure 2:**
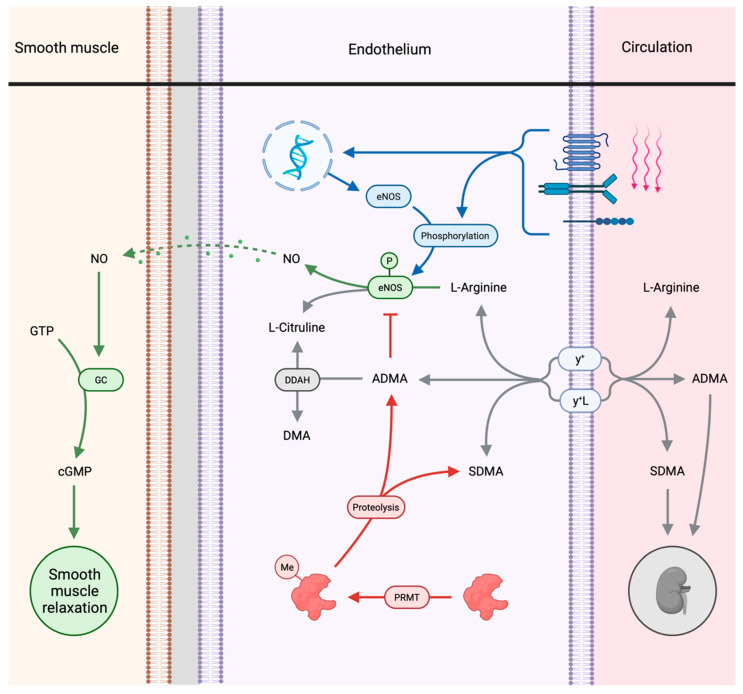
The figure shows the function of endothelial NO-synthase (eNOS) in endothelial cells. Blue pathways: eNOS is regulated transcriptionally and activated post-transcriptionally by serval intra-cellular pathways activated through mechanoreceptors that are sensitive to altered share forces from the luminal blood flow. These mechanoreceptors include integrins, vascular endothelial growth factor receptor-2, ion channels, G-protein-coupled receptors, and adhesion molecules such as platelet endothelial cell adhesion molecule-1. eNOS activity can also be modulated through cytokine, acetylcholine, and bradykinin receptors (not shown). Green pathway: Activated eNOS produces NO from L-arginine, with L-citrulline as a by-product. NO then diffuses to nearby cells and activates guanylate cyclase (GC) in the periarterial smooth muscle cells, which synthesize cyclic guanosine monophosphate (cGMP) from guanosine triphosphate (GTP). cGMP causes smooth muscle relaxation through several mechanisms. Red pathway: The activity of eNOS is inhibited by asymmetric dimethylarginine (ADMA) through competitive inhibition on the binding site for L-arginine on eNOS. SDMA has no direct effect on eNOS but indirectly reduces NO synthesis and L-arginine transport (not shown). L-arginine is an amino acid involved in protein synthesis and glycolysis (not shown), in addition to serving as a NO-precursor. Protein-bound L-arginine is methylated (Me) by protein arginine methyltransferases (PRMTs), and ADMA and SDMA are produced by proteolysis. Grey pathways: About 80% of intracellular ADMA is hydrolyzed by dimethylarginine dimethylaminohydrolase (DDAH) to L-citrulline and dimethylamine (DMA), while about 20% is transported to the blood and excreted in the kidneys. Most of SDMA is transported out of the cells and excreted in the kidneys. L-arginine, ADMA, and SDMA are transported bi-directionally through the y+ and y + L transporter systems. Created with BioRender.com.

The reduced arginine/ADMA ratio could explain our previous observation of a transient reduction in FMD in athletes immediately after completion of the Norseman Xtreme Triathlon [6]. FMD primarily depends on NO-mediated dilatation [10] and eNOS produces NO in endothelial cells [24,25]. The function of eNOS is illustrated and described in Figure 2, and as shown in the figure, L-arginine is the substrate for NO production [11,12] and ADMA is a competitive inhibitor that reduces the activity of eNOS [17,18]. A reduced L-arginine/ADMA ratio means less substrate relative to the competitive inhibitor, causing reduced activity of eNOS that could lead to less production of NO and, therefore, an impaired FMD response. 

It is important to emphasize that we have only measured levels of L-arginine and ADMA in serum, while the production of NO by eNOS is intracellular. L-arginine, ADMA, and SDMA are bidirectionally transported over the endothelial cell membrane by the y+ and y + L transporter systems [26]. In vitro studies on eNOS have demonstrated the importance of the differences between intracellular and extracellular L-arginine levels. Purified eNOS from bovine endothelial cells have a Km (concentration at which the reaction rate is half of its maximal value) of 3 μM [27], while the Km determined from extracellular levels of L-arginine on the activity of intracellular levels in bovine cells has been calculated to 29 μM [28]. In the same paper, the authors also demonstrated that the inhibition of L-arginine uptake reduces the activity of eNOS [28]. The intracellular levels of L-arginine and ADMA are therefore not necessarily reduced proportionally to the levels found in serum samples, due to possible alterations in the number of y+ and y + L transporters [29] and their activity [30]. However, in vitro studies have demonstrated a covariance of intracellular L-arginine with extracellular levels under physiological conditions, even in the presence of inhibitors [26,28]. We, therefore, find it probable that there are reduced intracellular levels of both L-arginine and ADMA after the race due to the significant reductions in serum levels by 31% and 16%, respectively. 

In the present study, we found increased levels of SDMA immediately after the race with continued increased levels the day after. In contrast to ADMA, SDMA has no direct effect on eNOS [17]. However, SDMA can indirectly reduce the eNOS activity by reducing intracellular L-arginine by competing for transport to the intracellular compartment over the y+ transport system in exchange for intracellular L-arginine [31]. Therefore, it is possible that the increased levels of circulatory SDMA could cause an even more considerable reduction in intracellular L-arginine after the race compared to the reduction observed in serum.

The exact mechanisms that cause exhaustion after prolonged exercise are still not completely elucidated. However several factors have been suggested, such as muscle damage [1,2,3,4,32], inflammation [1,2,3], exercise-induced bronchoconstriction [33], and reduction in essential nutrients [5,14,15]. The reduced levels of L-arginine and the L-arginine/ADMA ratio and the increased levels of SDMA observed after strenuous exercise, observed in the present study, may result in a state of reduced NO-synthesis capability. Therefore, it is plausible that part of the exhaustion after prolonged exercise is due to the reduced ability for vasodilatation, as we previously demonstrated by a blunted FMD response after an Ironman distance triathlon [6]. As L-arginine is a conditionally essential amino acid acquired through nutrition or synthesized from L-citrulline [34], it could be beneficial to increase L-arginine intake during or before prolonged exercise. Several studies have been conducted to test the effect of oral intake of L-arginine on performance, and a recent meta-analysis concluded that the oral intake of L-arginine could improve aerobic and anaerobic performance [35].

Reduced levels of L-arginine are also known to cause both pro-inflammatory and anti-inflammatory effects through effects upon macrophages, dendritic cells, and T-cells, as reviewed by Líndez and Reith [36]. It is also known that Ironman distance triathlons causes a state with increased circulatory cytokines, muscle damage markers, and leukocytosis [1,2,3,4]. Exercise-induced leukocytosis is believed to be of importance to muscle regeneration after muscle damage [37], and it is, therefore, possible that alterations in the leukocytes activity might affect recovery after prolonged exercise or diminish the immune system, making athletes more prone to disease. Therefore, we believe future studies should seek to investigate the leukocyte activity along with L-arginine levels after prolonged exercise to further elucidate the possible effects on the immune system. 

L-arginine trended toward normalized levels the day after the race, and we have previously found normalized levels one week after the same race [6]. ADMA increased beyond baseline levels the day after the race, and SDMA remained elevated compared to baseline the day after the race. We believe these increased ADMA and SDMA levels the day after the race may be due to increased proteolysis due to exercise-induced muscle damage and inflammation [38]. ADMA and SDMA could also have been increased due to reduced renal clearance as ADMA is partly excreted and SDMA is predominantly excreted through the kidneys [39], and we also know that athletes have increased serum creatinine after an Ironman distance triathlon [4]. We do not believe these changes are lasting, as exercise interventions in different populations have been shown to reduce ADMA [40,41] and SDMA [42]. However, we also found a continued reduced L-arginine/ADMA ratio the day after the race. A reduced L-arginine/ADMA ratio is considered a risk marker for atherosclerosis [22,43], and increased levels of ADMA and SDMA, as found the day after the race, are also risk factors for cardiovascular disease [22]. There is evidence of sub-clinical increased artery calcification and plaques in male long-distance runners [44,45]. Therefore, it would be of great interest in future studies to evaluate the time before normalization of the L-arginine/ADMA ratio, ADMA, and SDMA after an Ironman distance triathlon, as a limitation in the current study is that we only have measurements from before the race, at the finish line, and the first day after the race. In future studies, one should seek to measure the levels in the days and weeks following the race. It would also be of interest to follow athletes prospectively to study if these episodes of negatively affected biomarkers cause atherosclerotic changes in the arteries.

## 5. Conclusions

We found reduced levels of L-arginine and the L-arginine/ADMA ratio and increased SDMA upon completion of an Ironman distance triathlon. These altered biomarkers indicate a state of reduced NO synthesis capability and reduced endothelial function after the race. Furthermore, increased levels of ADMA and SDMA, and the reduced L-arginine/ADMA ratio the day after the race warrant further studies, as these biomarkers are known risk markers for atherosclerosis.

## Figures and Tables

**Table 1:** Characteristics of the subjects. Values are given as mean ± SD. N = 40; * Reported average weekly exercise last year.

Characteristic	Value
Age (years)	42 ± 8.5
Male (n)	33
Female (n)	7
Weight (kg)	77 ± 11
Height (m)	1.80 ± 0.08
Body mass index (kg/m^2^)	23.4 (22.1–24.7)
Weekly endurance exercise * (h)	14.0 ± 3.9
Weekly strength exercise * (h)	1.4 ± 1.6
Swim time (h)	1.2 ± 0.2
Bike time (h)	7.2 ± 1.0
Run time (h)	6.2 ± 1.0
Finish time (h)	14.5 ± 1.8

**Table 2:** Summary of all parameters given as mean ± SD.

Biomarker	Before Start	Finish Line	Day After
L-arginine (uM)	55.6 ± 8.8	38.3 ± 6.6	49.2 ± 9.6
ADMA (uM)	0.32 ± 0.05	0.27 ± 0.05	0.37 ± 0.05
SDMA (uM)	0.28 ± 0.03	0.43 ± 0.08	0.42 ± 0.09
L-arginine/ADMA ratio	55.6 ± 8.8	38.3 ± 6.6	49.2 ± 9.6

## Data Availability

The data presented in this study are openly available in the Supplemental Information as S1. Race times and the age of the subjects are omitted in the raw data to ensure anonymity for the subjects, as the race results are public.

## Supplementary Materials

The following are available online at https://www.mdpi.com/article/10.3390/sports9090120/s1, S1: Raw data. CSV file containing the raw data from the research.

## References

[1] Danielsson T., Carlsson J., Schreyer H., Ahnesjo J., Ten Siethoff L., Ragnarsson T., Tugetam A., Bergman P. (2017). Blood biomarkers in male and female participants after an Ironman-distance triathlon. PLoS ONE.

[2] Neubauer O., Konig D., Wagner K.H. (2008). Recovery after an Ironman triathlon: Sustained inflammatory responses and muscular stress. Eur. J. Appl. Physiol..

[3] Danielsson T., Schreyer H., Woksepp H., Johansson T., Bergman P., Mansson A., Carlsson J. (2019). Two-peaked increase of serum myosin heavy chain-alpha after triathlon suggests heart muscle cell death. BMJ Open Sport Exerc. Med..

[4] Nyborg C., Melau J., Bonnevie-Svendsen M., Mathiasen M., Melsom H.S., Storsve A.B., Hisdal J. (2020). Biochemical markers after the Norseman Extreme Triathlon. PLoS ONE.

[5] Storsve A.B., Johnsen L., Nyborg C., Melau J., Hisdal J., Burri L. (2020). Effects of Krill Oil and Race Distance on Serum Choline and Choline Metabolites in Triathletes: A Field Study. Front. Nutr..

[6] Nyborg C., Melsom H.S., Bonnevie-Svendsen M., Melau J., Seljeflot I., Hisdal J. (2021). Transient Reduction of FMD-Response and L-arginine Accompanied by Increased Levels of E-Selectin, VCAM, and ICAM after Prolonged Strenuous Exercise. Sports.

[7] Celermajer D.S., Sorensen K.E., Bull C., Robinson J., Deanfield J.E. (1994). Endothelium-dependent dilation in the systemic arteries of asymptomatic subjects relates to coronary risk factors and their interaction. J. Am. Coll. Cardiol..

[8] Pohl U., Holtz J., Busse R., Bassenge E. (1986). Crucial role of endothelium in the vasodilator response to increased flow in vivo. Hypertension.

[9] Li Y.S., Haga J.H., Chien S. (2005). Molecular basis of the effects of shear stress on vascular endothelial cells. J. Biomech..

[10] Green D.J., Dawson E.A., Groenewoud H.M., Jones H., Thijssen D.H. (2014). Is flow-mediated dilation nitric oxide mediated?: A meta-analysis. Hypertension.

[11] Zhao Y., Vanhoutte P.M., Leung S.W. (2015). Vascular nitric oxide: Beyond eNOS. J. Pharmacol. Sci..

[12] Schmidt H.H., Nau H., Wittfoht W., Gerlach J., Prescher K.E., Klein M.M., Niroomand F., Bohme E. (1988). Arginine is a physiological precursor of endothelium-derived nitric oxide. Eur. J. Pharmacol..

[13] Haralambie G., Berg A. (1976). Serum urea and amino nitrogen changes with exercise duration. Eur. J. Appl. Physiol. Occup. Physiol..

[14] Cuisinier C., Ward R.J., Francaux M., Sturbois X., de Witte P. (2001). Changes in plasma and urinary taurine and amino acids in runners immediately and 24h after a marathon. Amino Acids.

[15] Schader J.F., Haid M., Cecil A., Schoenfeld J., Halle M., Pfeufer A., Prehn C., Adamski J., Nieman D.C., Scherr J. (2020). Metabolite Shifts Induced by Marathon Race Competition Differ between Athletes Based on Level of Fitness and Performance: A Substudy of the Enzy-MagIC Study. Metabolites.

[16] Bedford M.T., Clarke S.G. (2009). Protein arginine methylation in mammals: Who, what, and why. Mol. Cell.

[17] Vallance P., Leone A., Calver A., Collier J., Moncada S. (1992). Accumulation of an endogenous inhibitor of nitric oxide synthesis in chronic renal failure. Lancet.

[18] Vallance P., Leiper J. (2002). Blocking NO synthesis: How, where and why?. Nat. Rev. Drug Discov..

[19] Notsu Y., Yano S., Shibata H., Nagai A., Nabika T. (2015). Plasma arginine/ADMA ratio as a sensitive risk marker for atherosclerosis: Shimane CoHRE study. Atherosclerosis.

[20] Bode-Boger S.M., Scalera F., Kielstein J.T., Martens-Lobenhoffer J., Breithardt G., Fobker M., Reinecke H. (2006). Symmetrical dimethylarginine: A new combined parameter for renal function and extent of coronary artery disease. J. Am. Soc. Nephrol..

[21] Strobel J., Mieth M., Endress B., Auge D., Konig J., Fromm M.F., Maas R. (2012). Interaction of the cardiovascular risk marker asymmetric dimethylarginine (ADMA) with the human cationic amino acid transporter 1 (CAT1). J. Mol. Cell. Cardiol..

[22] Schlesinger S., Sonntag S.R., Lieb W., Maas R. (2016). Asymmetric and Symmetric Dimethylarginine as Risk Markers for Total Mortality and Cardiovascular Outcomes: A Systematic Review and Meta-Analysis of Prospective Studies. PLoS ONE.

[23] Wolyniec W., Kasprowicz K., Giebultowicz J., Korytowska N., Zorena K., Bartoszewicz M., Rita-Tkachenko P., Renke M., Ratkowski W. (2019). Changes in Water Soluble Uremic Toxins and Urinary Acute Kidney Injury Biomarkers After 10- and 100-km Runs. Int. J. Environ. Res. Public Health.

[24] Palmer R.M., Ashton D.S., Moncada S. (1988). Vascular endothelial cells synthesize nitric oxide from L-arginine. Nature.

[25] Garcia V., Sessa W.C. (2019). Endothelial NOS: Perspective and recent developments. Br. J. Pharmacol..

[26] Sala R., Rotoli B.M., Colla E., Visigalli R., Parolari A., Bussolati O., Gazzola G.C., Dall’Asta V. (2002). Two-way arginine transport in human endothelial cells: TNF-alpha stimulation is restricted to system y(+). Am. J. Physiol. Cell Physiol..

[27] Pollock J.S., Forstermann U., Mitchell J.A., Warner T.D., Schmidt H.H., Nakane M., Murad F. (1991). Purification and characterization of particulate endothelium-derived relaxing factor synthase from cultured and native bovine aortic endothelial cells. Proc. Natl. Acad. Sci. USA.

[28] Hardy T.A., May J.M. (2002). Coordinate regulation of L-arginine uptake and nitric oxide synthase activity in cultured endothelial cells. Free Radic. Biol. Med..

[29] Cui H., Chen B., Chicoine L.G., Nelin L.D. (2011). Overexpression of cationic amino acid transporter-1 increases nitric oxide production in hypoxic human pulmonary microvascular endothelial cells. Clin. Exp. Pharmacol. Physiol..

[30] Arancibia-Garavilla Y., Toledo F., Casanello P., Sobrevia L. (2003). Nitric oxide synthesis requires activity of the cationic and neutral amino acid transport system y+L in human umbilical vein endothelium. Exp. Physiol..

[31] Closs E.I., Basha F.Z., Habermeier A., Forstermann U. (1997). Interference of L-arginine analogues with L-arginine transport mediated by the y+ carrier hCAT-2B. Nitric Oxide.

[32] Del Coso J., Gonzalez-Millan C., Salinero J.J., Abian-Vicen J., Soriano L., Garde S., Perez-Gonzalez B. (2012). Muscle damage and its relationship with muscle fatigue during a half-iron triathlon. PLoS ONE.

[33] Stensrud T., Rossvoll O., Mathiassen M., Melau J., Illidi C., Ostgaard H.N., Hisdal J., Stang J. (2020). Lung function and oxygen saturation after participation in Norseman Xtreme Triathlon. Scand. J. Med. Sci. Sports.

[34] Tapiero H., Mathe G., Couvreur P., Tew K.D. (2002). I. Arginine. Biomed. Pharmacother..

[35] Viribay A., Burgos J., Fernandez-Landa J., Seco-Calvo J., Mielgo-Ayuso J. (2020). Effects of Arginine Supplementation on Athletic Performance Based on Energy Metabolism: A Systematic Review and Meta-Analysis. Nutrients.

[36] Marti I.L.A.A., Reith W. (2021). Arginine-dependent immune responses. Cell. Mol. Life Sci..

[37] MacIntyre D.L., Reid W.D., Lyster D.M., Szasz I.J., McKenzie D.C. (1996). Presence of WBC, decreased strength, and delayed soreness in muscle after eccentric exercise. J. Appl. Physiol..

[38] Cerqueira E., Marinho D.A., Neiva H.P., Lourenco O. (2019). Inflammatory Effects of High and Moderate Intensity Exercise-A Systematic Review. Front. Physiol..

[39] McDermott J.R. (1976). Studies on the catabolism of Ng-methylarginine, Ng, Ng-dimethylarginine and Ng, Ng-dimethylarginine in the rabbit. Biochem. J..

[40] Gomes V.A., Casella-Filho A., Chagas A.C., Tanus-Santos J.E. (2008). Enhanced concentrations of relevant markers of nitric oxide formation after exercise training in patients with metabolic syndrome. Nitric Oxide.

[41] Mittermayer F., Pleiner J., Krzyzanowska K., Wiesinger G.F., Francesconi M., Wolzt M. (2005). Regular physical exercise normalizes elevated asymmetrical dimethylarginine concentrations in patients with type 1 diabetes mellitus. Wien. Klin. Wochenschr..

[42] Riccioni G., Scotti L., Guagnano M.T., Bosco G., Bucciarelli V., Di Ilio E., Speranza L., Martini F., Bucciarelli T. (2015). Physical exercise reduces synthesis of ADMA, SDMA, and L-Arg. Front. Biosci..

[43] Brinkmann S.J., Worner E.A., Buijs N., Richir M., Cynober L., van Leeuwen P.A., Couderc R. (2015). The Arginine/ADMA Ratio Is Related to the Prevention of Atherosclerotic Plaques in Hypercholesterolemic Rabbits When Giving a Combined Therapy with Atorvastatine and Arginine. Int. J. Mol. Sci..

[44] Merghani A., Maestrini V., Rosmini S., Cox A.T., Dhutia H., Bastiaenan R., David S., Yeo T.J., Narain R., Malhotra A. (2017). Prevalence of Subclinical Coronary Artery Disease in Masters Endurance Athletes With a Low Atherosclerotic Risk Profile. Circulation.

[45] Mohlenkamp S., Lehmann N., Breuckmann F., Brocker-Preuss M., Nassenstein K., Halle M., Budde T., Mann K., Barkhausen J., Heusch G. (2008). Running: The risk of coronary events: Prevalence and prognostic relevance of coronary atherosclerosis in marathon runners. Eur. Heart J..

